# Congenital Zika syndrome arising from sexual transmission of Zika virus, a case report

**DOI:** 10.1186/s40738-018-0053-5

**Published:** 2019-01-03

**Authors:** Christina D. Yarrington, Davidson H. Hamer, Wendy Kuohung, Aviva Lee-Parritz

**Affiliations:** 1 Department of Obstetrics and Gynecology, Division of Maternal & Fetal Medicine, Boston Medical Center, Boston, MA 02118 USA; 2 Boston Medical Center Department of Medicine, Section of Infectious Diseases, Boston University School of Public Health Department of Global Health and Center for Global Health and Development, Boston, MA USA; 3 Department of Obstetrics and Gynecology, Division of Reproductive Endocrinology and Infertility, Boston Medical Center, Boston, MA 02118 USA

**Keywords:** Zika, Congenital zika syndrome, Sexually transmitted infection, Microcephaly

## Abstract

**Background:**

Sexual transmission of Zika virus is well documented and pregnant women are advised to abstain or use barrier protection if a sexual partner has risk for Zika infection. However, to date there has not been a documented case of the congenital Zika syndrome resulting from sexual transmission.

**Case presentation:**

A 32 year-old woman who had not traveled to any area with local Zika transmission in years became pregnant via frozen embryo transfer. Her husband traveled to Haiti several times prior to embryo transfer and during the pregnancy. Neither partner was ever symptomatic. In her second trimester when recommendations were published by the Centers for Disease Control and Prevention (CDC) regarding prevention of sexual transmission during pregnancy she was counseled to abstain or use barrier protection with her partner. At delivery, the infant head circumference measured less than the first percentile. Placental samples were sent to the CDC and all were positive for Zika RNA by RT-PCR. Evaluation for other causes of microcephaly was negative. Consistent with the most up to date diagnostic parameters for congenital Zika, including viral infection of the placenta, the baby was diagnosed with congenital Zika syndrome.

**Conclusions:**

Transmission via sexual contact during assisted reproductive therapies (ART) and pregnancy can result in Zika fetopathy. This case supports recommendations to counsel women undergoing ART and pregnant women to use barrier protection with partners with Zika exposure regardless of their symptoms.

## Background

In February 2016, as an increasing series of sexually transmitted Zika virus cases were identified, the CDC issued a Health Alert Network Advisory with guidelines for preventing sexual transmission of Zika virus [1, 2]. Since then numerous case reports and series have demonstrated male-to-female, male-to-male, and female-to-male sexual transmission of Zika virus [3]. Despite the logical connection of potential infection of a woman from a male partner and subsequent vertical transmission in pregnancy, there has not to date been a case of congenital Zika syndrome resulting from sexual transmission of the virus. We describe the first baby with microcephaly from Zika virus vertically acquired after sexual transmission during assisted reproductive therapy via frozen embryo transfer and pregnancy.

## Case presentation

A 32 year old woman, gravida three para one with a history of cervical incompetence, polycystic ovarian syndrome, antiphospholipid antibody syndrome (APLS), and tubal factor infertility, conceived with frozen embryo transfer of a single blastocyst resulting from standard insemination technique; intracellular sperm injection was not performed. They did not opt for preimplantation genetic screening. The embryo was originally frozen in 2013, 2 years before the first identification of a Zika case in Haiti. Embryo transfer occurred in April of 2016. Her husband was actively traveling back and forth to Haiti for work before and during the pregnancy.

Her history of two mid-trimester losses was managed with an abdominal cerclage placed pre-pregnancy. She was treated with prophylactic low molecular weight heparin for APLS and insulin for her type two diabetes mellitus. She received betamethasone in the early third trimester for an episode of threated preterm labor. At delivery she was euglycemic.

Endemic Zika was identified in Haiti per the CDC early in the epidemic [4]. When recommendations were issued regarding the possibility of sexual transmission of Zika virus in August 2016, her maternal fetal medicine provider counseled her to use condoms or refrain from intercourse with her husband, whose business travel continued through the pregnancy [5]. She herself never left the greater Boston area prior to or during the pregnancy. She had not been to her native Haiti in over 10 years. There has never been any local transmission of Zika virus in the state of Massachusetts. Additionally, interview with the couple after delivery confirmed that neither of them ever experienced any symptoms of Zika infection. In the absence of symptoms in either partner, it was not our practice to recommend Zika serology during the pregnancy to screen for sexual exposure.

The patient underwent extensive fetal surveillance because of her multiple morbidities. She had a level II fetal survey at 18 weeks that revealed normal intracranial anatomy and head circumference (HC), and occipitofrontal diameter (OFD) measuring only 2 days smaller than her best dates. Biometry performed at 29 and 33 weeks was normal, and neither the HC nor the OFD measured less than 5%ile for gestational age. There was never any evidence of intracranial calcifications, ventriculomegaly, or abnormal posturing on antenatal ultrasound. She delivered in the 37th week via scheduled cesarean section. Her baby boy had APGARs of 8 (− 2 for color) and 9 (− 1 for color) at 1 and 5 min. He weighed 2775 g (30%ile by Fenton curve), was 49.5 cm long (65%ile) and had a head circumference of 29.2 cm (0%ile). The placenta was sent for conventional pathologic analysis given the maternal comorbidities. In addition, in light of the small measured neonatal HC and possible Zika virus sexual exposure, samples were sent to the CDC for evaluation. The timing of the IVF cycle relative to the Zika epidemic in Haiti was discussed with the Massachusetts Department of Public Health and CDC at length and the frozen embryo was determined not to be the source of infection.

The baby had a normal hearing screen and was discharged on day of life (DOL) 6. His workup for microcephaly included serum and urine Zika RT-PCR and IgM, both of which were ultimately negative; CMV, head ultrasound and MRI were also negative. Head ultrasound performed in the first week of life was notable for bilateral mineralizing vasculopathy but no intraparenchymal calcifications and otherwise normal anatomy. A subsequent head MRI was normal. The mother was rubella immune and had negative testing for other relevant TORCH infections. Approximately 3 months after delivery, confirmation was received from the CDC that all placental samples were positive for Zika RNA, thus supporting the diagnosis of congenital Zika syndrome. By the time the placental results from the CDC had been received, both parents were too far removed from the time of suspected infection to be able to do serology. HC at a pediatric visit shortly after receipt of the CDC report revealed an interval increase in HC, although it was still less than third percentile for his age. The child continues to meet normal pediatric milestones and receives early intervention services as well as assessment by pediatric neurodevelopment specialists. A genetics evaluation will be pursued if there is any lag in his neurodevelopment.

## Discussion

Sexual transmission of Zika virus was first described in 2008 but became a more pressing public health issue in the wake of the epidemic of 2016 and the associated rise in microcephaly [6]. Given the known teratogenicity of maternal viral infection, pregnant women are advised to avoid unprotected sexual contact during pregnancy with a partner with possible exposure to the virus [7]. Additionally, the finding that live cultivable virus and viral particles have been identified in semen samples up to 69 and 188 days respectively after index infection informed earlier recommendations to avoid conception or fertility treatment for a minimum of 6 months after possible exposure for a male partner [3]. However, the most recent recommendations from the CDC to avoid sexual transmission have reduced the recommended interval to 2 months after known infection or last exposure to Zika virus for women and 3 months for men [8].

There are two prior case reports of sexual transmission with an asymptomatic male partner [9, 10]. In our case, sexual transmission occurred from an asymptomatic man to his partner resulting in placental passage and fetal infection despite asymptomatic maternal infection. While most clinicians at this point are comfortable directing their patients to avoid travel immediately prior to and during pregnancy, counseling a woman to avoid unprotected intercourse with her partner during pregnancy can be a more difficult conversation. Until now, the rationale to advise protection during coitus if a partner has possible Zika exposure had been based on the logical connection of known and accepted modes of infection, e.g. mosquito bite followed by sexual contact. However, there had not yet been any real-world proof that transvaginal infection could lead to fetal infection in humans. Our case illustrates a serologically positive infant with microcephaly born to a woman who had not traveled in years and whose only possible route of infection was sexual contact. The child’s reassuring neurologic development in his first months of life reflects the wide range of phenotypes seen in congenital Zika infection [11].

Our case is also the first to report Zika fetopathy in a pregnancy resulting from a frozen embryo transfer. Our patient had 5 day 5 embryos frozen after a fresh in vitro fertilization cycle in 2013. The patient presented in late February of 2016 with her partner, who had been in Haiti for the 5 months prior, to sign consents for frozen embryo transfer, and a high-quality blastocyst was transferred in mid-April 2016. Therefore, sexual transmission of ZIKV may have occurred just before or after embryo transfer or later in the pregnancy. There is one reported case of congenital Zika syndrome in a pregnancy conceived through in vitro fertilization with fresh embryo transfer in Venezuela [12]. The woman developed symptoms at 10 weeks of gestation and had positive RT-PCR for ZIKV at that time. The pregnancy was ultimately terminated after multiple features of congenital Zika syndrome were identified on ultrasound and confirmed with amniocentesis. It is impossible to know the gestational age of infection as there is a wide range of phenotypes from infections in all three trimesters and not all cases are identified on antenatal ultrasound [11].

This case also highlights the importance of paired epidemiology of tracking maternal infection and birth defects related to Zika infection. Identifying infants at risk for congenital Zika syndrome has required a paradigm shift to coordinate pregnancy and birth defects surveillance. Continued coordinated surveillance is particularly important in light of the limitations of available serology. While a woman who presents more than 12 weeks after possible exposure cannot have direct serology performed, laboratory evidence of Zika infection in pregnancy can be established by placental Zika RNA. Additionally, as in our case, some infants with congenital Zika syndrome test negative for the virus at delivery [13, 14]. The common observation of waning level of Zika RNA and IgM or a lack of IgM response in the fetus is cited in the latest publication from the CDC on diagnostic parameters for congenital Zika syndrome where they include placental Zika RNA as a possible diagnostic criterion for congenital infection [14]. The limitations of fetal serology underscores the importance of tracking antenatal findings associated with the congenital Zika syndrome to identify placentae as well as infants that need further evaluation at delivery.

As awareness and diagnostic tools continue to improve, we may continue to see an increase in the number of infants with consequences of sexually transmitted Zika. Efforts to link antenatal and postnatal findings to maternal risk of exposure will be key to identifying infants at risk. In addition, continued vigilance in prenatal counseling about barrier protection or abstinence during assisted reproductive therapies when a male partner has exposure risk will reduce the risk of congenital Zika syndrome.
